# 2553. Safety, tolerability and pharmacokinetics of HS-10366 in healthy Chinese subjects: A randomized, double-blind, placebo-controlled phase 1 trial

**DOI:** 10.1093/ofid/ofad500.2170

**Published:** 2023-11-27

**Authors:** Xiaoyan Liu, Rui Zhang, Rong Li, Qiong Wu, Chao Pan, Xiangqing Yu, Wen Jia, Peng Xia, Yuhui Liu, Benjie Wang, Shuwen Yu

**Affiliations:** Phase 1 Clinical Trial Center, Qilu Hospital of Shandong University; NMPA Key Laboratory for Clinical Research and Evaluation of Innovative Drugs, Jinan, Shandong, China; Phase 1 Clinical Trial Center, Qilu Hospital of Shandong University; NMPA Key Laboratory for Clinical Research and Evaluation of Innovative Drugs, Jinan, Shandong, China; Phase 1 Clinical Trial Center, Qilu Hospital of Shandong University; NMPA Key Laboratory for Clinical Research and Evaluation of Innovative Drugs, Jinan, Shandong, China; Jiangsu Hansoh Pharmaceutical Group Co., Ltd., Shanghai, Shanghai, China; Jiangsu Hansoh Pharmaceutical Group Co., Ltd., Shanghai, Shanghai, China; Jiangsu Hansoh Pharmaceutical Group Co., Ltd., Shanghai, Shanghai, China; Jiangsu Hansoh Pharmaceutical Group Co., Ltd., Shanghai, Shanghai, China; Jiangsu Hansoh Pharmaceutical Group Co., Ltd., Shanghai, Shanghai, China; Jiangsu Hansoh Pharmaceutical Group Co., Ltd., Shanghai, Shanghai, China; Phase 1 Clinical Trial Center, Qilu Hospital of Shandong University; NMPA Key Laboratory for Clinical Research and Evaluation of Innovative Drugs, Jinan, Shandong, China; Phase 1 Clinical Trial Center, Qilu Hospital of Shandong University; NMPA Key Laboratory for Clinical Research and Evaluation of Innovative Drugs, Jinan, Shandong, China

## Abstract

**Background:**

HS-10366 (generic name: ibrexafungerp) is a first-in-class and orally active triterpenoid antifungal agent with broad antifungal activity against *Candida* spp, *Aspergillus* spp, and other fungal pathogens. It was approved by U.S. Food and Drug Administration for the treatment of vulvovaginal candidiasis. The study aimed to evaluate the safety, tolerability and pharmacokinetic characteristics of oral HS-10366 in healthy Chinese adults.

Plasma HS-10366 concentration (Mean ± SD, log plots) time profiles after a single dose.

**Figure:**
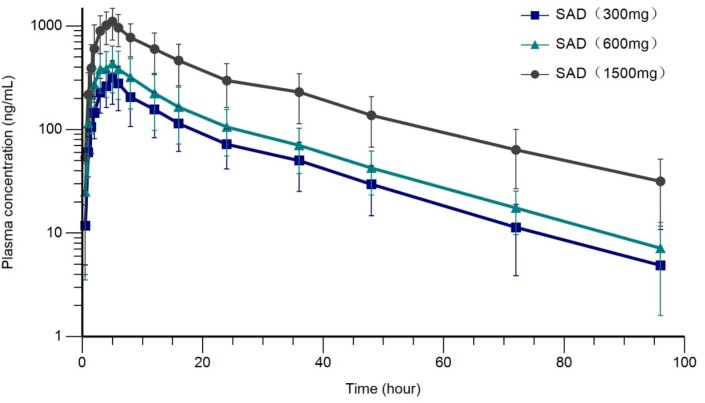

SAD: single ascending dose.

Plasma HS-10366 concentration (Mean ± SD, log plots) time curve after repeated doses.

**Figure:**
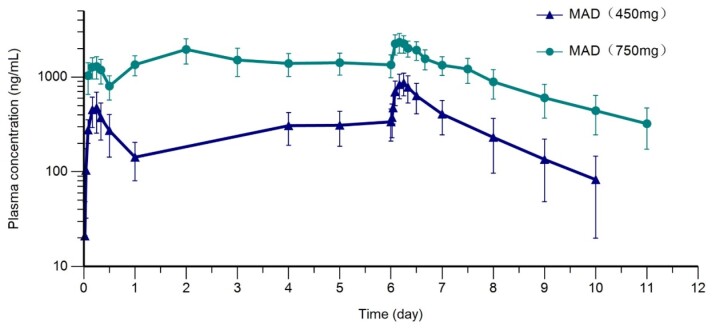

MAD: multiple ascending doses.

**Methods:**

A single-center, randomized, double-blind, placebo-controlled single ascending dose (SAD, n=42) and multiple ascending dose (MAD, n=28) study was conducted in healthy Chinese subjects from March to October 2022. There were three cohorts in SAD stage (300, 600 and 1500 mg) and two cohorts in MAD stage (450 mg QD for 7 days; a loading dose as 750 mg BID for first 2 days followed by a maintenance dose of 750 mg QD for consecutive 5 days). Eligible participants in each cohort were randomly assigned in a 6:1 ratio to receive either HS-10366 or placebo orally. The primary objectives were to evaluate the safety and tolerability. The secondary objectives were to evaluate pharmacokinetic parameters mainly including C_max_, AUC and T_1/2_.

**Results:**

A total of 70 healthy Chinese subjects were enrolled in the study. Mean (SD) age was 29.0 (6.32) and 55.7% were male. All treatment-emergent adverse events (TEAEs) were mild or moderate. There were no serious adverse events, no subjects were discontinued due to TEAEs, and all TEAEs were recovered or resolved. The most common TEAEs were diarrhea, abdominal pain and nausea in both SAD and MAD. In SAD stage, C_max_ and AUC increased in an approximately dose-proportional manner in the dose range of 300-1500 mg. T_1/2_ was within 18.29-21.30 hours. In MAD stage, the accumulation ratio for C_max_ and AUC indicated a mild to moderate accumulation after continuous dosing.

Demographic and clinical characteristics of subjects in SAD stage.

**Figure:**
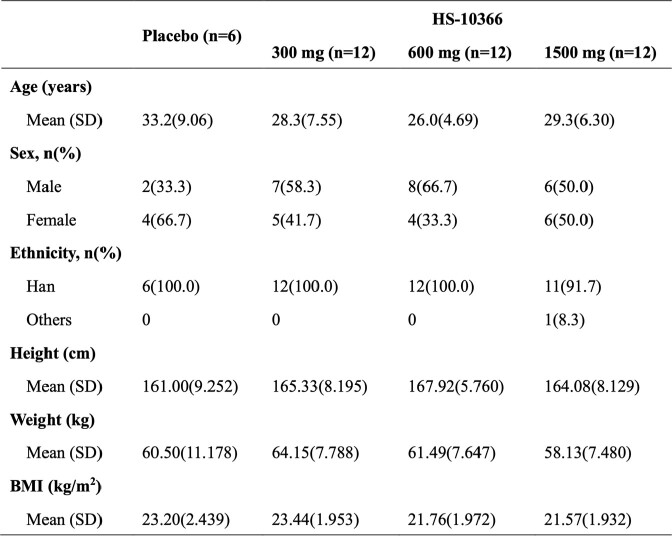

Demographic and clinical characteristics of subjects in MAD stage.

**Figure:**
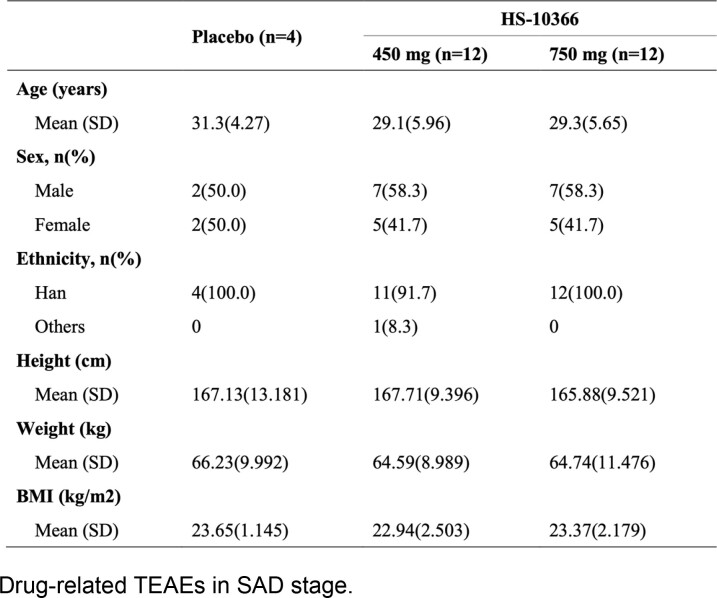

Drug-related TEAEs in SAD stage.

**Figure:**
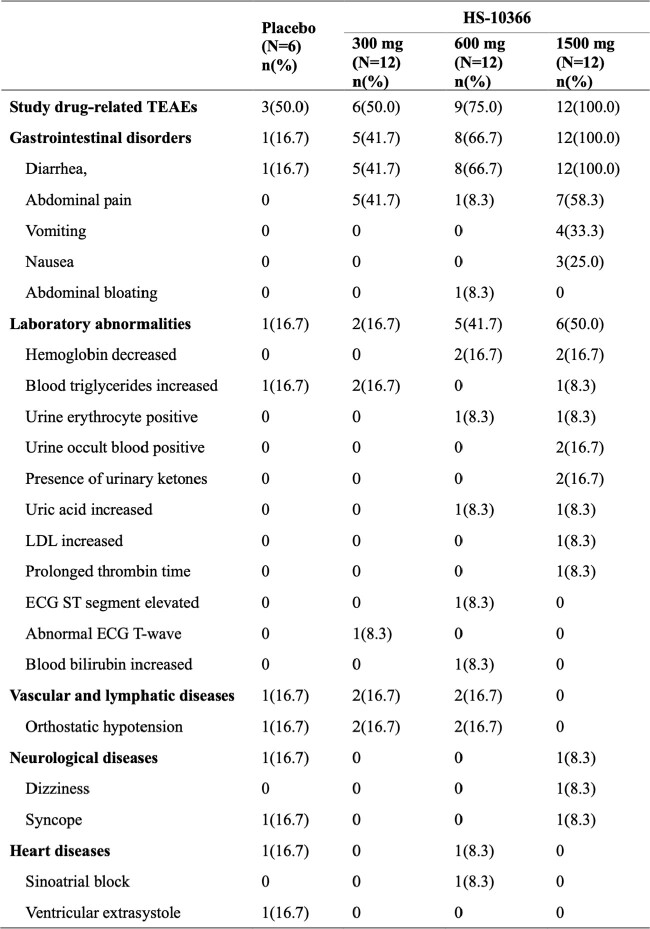

**Conclusion:**

This phase 1 study demonstrates a favorable safety, tolerability and PK profile of HS-10366 in healthy Chinese subjects.

Drug-related TEAEs in MAD stage.

**Figure:**
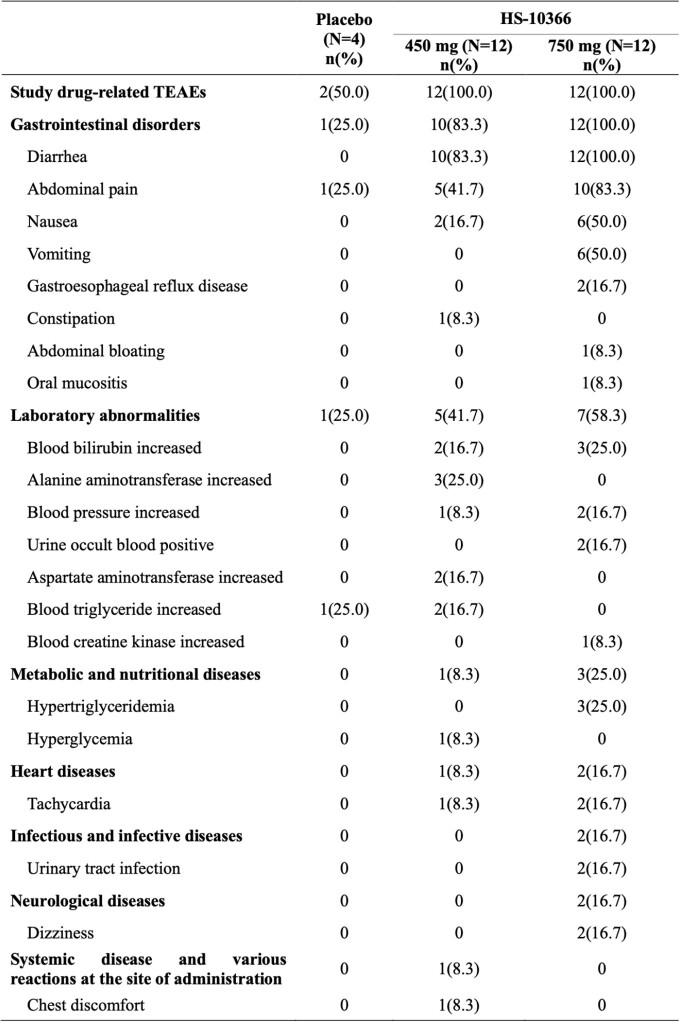

The percentage was calculated using the number of subjects in each dose group of the safety analysis set as the denominator. Adverse events associated with the study drug were defined as those in which the association with the study drug during administration was “definitely relevant”, “probably relevant”, or “undetermined”. Adverse events were coded based on MedDRA 25.1.

Descriptive summary of pharmacokinetic parameters of HS-10366 in single ascending dose stage (fasting).

**Figure:**
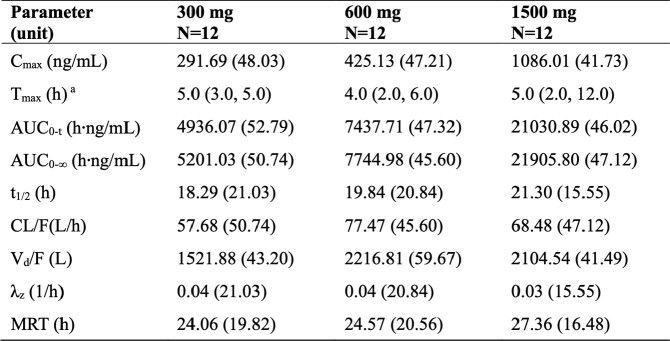

Parameters were shown as Geometric Mean (geometric coefficient of variability %), unless otherwise noted. aMedian (minimum, maximum). Abbreviations: N=number of subjects, Cmax=maximum observed plasma concentration, Tmax=time of Cmax, AUC0-t=area under the concentration time curve from time 0 to the last quantifiable time, AUC0-∞=area under the concentration time curve from time 0 extrapolate to infinity, t1/2=terminal half-life, CL/F=apparent clearance, Vd/F=apparent volume of distribution, λz=terminal rate constant, MRT= mean residence time.

Descriptive summary of pharmacokinetic parameters of HS-10366 in MAD stage.

**Figure:**
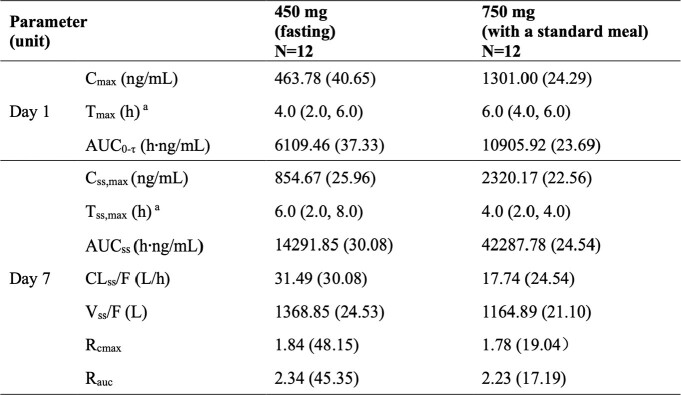

Parameters were shown as Geometric Mean (geometric coefficient of variability %), unless otherwise noted. aMedian (minimum, maximum). Abbreviations: N=number of subjects, Cmax=maximum observed plasma concentration, Tmax=time of Cmax, AUC0-τ=area under the concentration time curve during dosing interval (the dosing interval of 450 mg group and 750 mg group is 24h and 12h in day 1, respectively), Css,max=the maximum concentration at steady state, Tss,max=time of the maximum concentration at steady state, AUCss=area under the concentration- time curve at steady state, CLss/F=apparent clearance at steady state, Vss/F=apparent volume of distribution at steady state, Rcmax=accumulation ratio of Cmax, Rauc= accumulation ratio of AUC (for 750 mg group, Rauc=AUC0-12,day7/AUC0-τ).

**Disclosures:**

**Qiong Wu, MD**, Jiangsu Hansoh Pharmaceutical Group Co. Ltd: Board Member **Chao Pan, MD**, Jiangsu Hansoh Pharmaceutical Group Co. Ltd: Employee of the company **Xiangqing Yu, PhD**, Jiangsu Hansoh Pharmaceutical Group Co. Ltd: Employee of the company **Wen Jia, MD**, Jiangsu Hansoh Pharmaceutical Group Co. Ltd: Employee of the company **Peng Xia, MD**, Jiangsu Hansoh Pharmaceutical Group Co. Ltd: Employee of the company **Yuhui Liu, PhD**, Jiangsu Hansoh Pharmaceutical Group Co. Ltd: Employee of the company

